# Design and Development of nEMoS, an All-in-One, Low-Cost, Web-Connected and 3D-Printed Device for Environmental Analysis

**DOI:** 10.3390/s150613012

**Published:** 2015-06-04

**Authors:** Francesco Salamone, Lorenzo Belussi, Ludovico Danza, Matteo Ghellere, Italo Meroni

**Affiliations:** Construction Technologies Institute, National Research Council of Italy (ITC-CNR), Via Lombardia, 49, 20098 San Giuliano Milanese (MI), Italy; E-Mails: belussi@itc.cnr.it (L.B.); danza@itc.cnr.it (L.D.); ghellere@itc.cnr.it (M.G.); meroni@itc.cnr.it (I.M.)

**Keywords:** indoor air quality, thermal comfort, Arduino, App Inventor, Internet of Things, Indoor Environmental Quality, environmental monitoring system

## Abstract

The Indoor Environmental Quality (IEQ) refers to the quality of the environment in relation to the health and well-being of the occupants. It is a holistic concept, which considers several categories, each related to a specific environmental parameter. This article describes a low-cost and open-source hardware architecture able to detect the indoor variables necessary for the IEQ calculation as an alternative to the traditional hardware used for this purpose. The system consists of some sensors and an Arduino board. One of the key strengths of Arduino is the possibility it affords of loading the script into the board’s memory and letting it run without interfacing with computers, thus granting complete independence, portability and accuracy. Recent works have demonstrated that the cost of scientific equipment can be reduced by applying open-source principles to their design using a combination of the Arduino platform and a 3D printer. The evolution of the 3D printer has provided a new means of open design capable of accelerating self-directed development. The proposed **n**ano **E**nvironmental **Mo**nitoring **S**ystem (nEMoS) instrument is shown to have good reliability and it provides the foundation for a more critical approach to the use of professional sensors as well as for conceiving new scenarios and potential applications.

## 1. Introduction

The importance of well-being and satisfaction of the building users is an imperative objective and an always open challenge for the actors of the building sector. Several studies have been made over the years aimed at analyzing both the interaction between the energy performances and the indoor quality of buildings [1,2,3,4,5] and providing objective methods to allow the parallel growth of these components [6,7]. A literature review highlights the necessity to continuously monitor and improve the indoor conditions and energy performance of buildings [8].

A person is generally in a state of well-being when he does not feel any sense of discomfort and he is therefore in a condition of absolute neutrality with respect to the surrounding environment. The IEQ is the aggregation of a set of different categories of comfort, each of which refers to a specific environmental parameter: Indoor Air Quality (IAQ); Indoor thermal Comfort Quality (ICQ); Indoor Lighting Quality (ILQ); Indoor Sound Quality (ISQ). Specific performance indicators are determined for each category allowing the assessment of the level of specific and absolute comfort.

The importance of the IEQ and of the devices able to guarantee the best indoor conditions is well established, especially with the new Zero Energy Building (ZEB) concept [9]. One of the challenges in the IEQ market is the real-time monitoring of the indoor conditions aimed at allowing the best management practices, even from an energy saving perspective. One of the end-user markets with a high potential is represented by the residential dwellings. The peculiarity of this market requires a series of measures for the diffusion of these practices, first of all the simplicity and the non-invasiveness of the devices. Moving from these premises the present work presents a simple, accurate, and easy to use device based on an open hardware/software concept and aimed at evaluating the IEQ.

The monitoring systems for whole building assessment have enjoyed an important impulse recently, especially thanks to the development of wireless equipment allowing a more accurate and less expensive detection of the environmental variables [10]. In recent years, the development of open hardware microcontrollers has allowed the production of low cost monitoring systems [11,12].

The evaluation of the IEQ requires the use of tools able to detect specific environmental variables [13]. Following the so-called *Internet of Things* approach, that has allowed the web to evolve from the static web pages of the 90s to the web 2.0 (social networking web) of the 2000 s and up to the web 3.0 (ubiquitous computing web) of the present day [14,15,16,17], a specific device has been built.

Exploiting the huge potential of these tools and the wide availability of sensors, this article describes an “all-in-one” device, called **n**ano **E**nvironmental **Mo**nitoring **S**ystem (nEMoS), aimed at assessing the Indoor Environmental Quality (IEQ) of buildings. The two features on which nEMoS is based are the inexpensiveness and the consistency of the detected data. For the former of these purposes only low cost sensors and microcontrollers have been chosen and for the latter the detected data have been compared with those of typical commercial sensors. The paper is thus focused on a comparison between nEMoS device and commercial equipment.

All sensors are put in a controlled environment and calibrated thus defining the appropriate correction algorithms. The implementation of the device nEMoS required a preliminary assessment aimed at identifying:

- type of microcontroller;
- data connection type;
- design and construction of the case.

Several manufacturers such as Parallax Inc., Coridium Corporation, FTDI, Picaxe, Arduino, as well as many others, have proposed quite popular solutions. All of these boards are inexpensive. However, Arduino boards offer one critical advantage: the open source philosophy (both hardware and software), which capitalizes on the massive non-expert community that has flourished around the Arduino concept. A very rough estimate of the community size can be gleaned from a Google search reporting more than 38 million hits for “Arduino”. In fact, the large user base and the growing market have shown an increasing interest around the Arduino concept.

The collected data are sent to a cloud server for storage, thanks to the Wi-Fi shield mounted on the nEMoS. The Wi-Fi shield allows one to connect nEMoS to the internet using the 802.11 protocol. It is based on the HDG204 Wireless LAN 802.11b/g System in-Package.

For the implementation of the case, a 3D printer (PowerWasp, WASProject a project of CSP s.r.l., Massa Lombarda (RA), Italy) has been applied. This 3D printer implements the fused deposition modeling (FDM) technology and uses polylactide (PLA) for printing. PLA is one of the most eco-friendly 3D printing materials available, being made from annually renewable resources (corn starch) and requiring less energy to process than traditional (petroleum-based) plastics. Besides 3D printing, PLA is often used in food containers, such as candy wrappers, and biodegradable medical implants, such as sutures.

## 2. Experimental Section

The fundamental core of the system is a microcontroller released as an open hardware Arduino PCB [18]. The word Arduino refers to PCBs that differ in size, power, available analog and digital pins and intended use that are programmable through a programming language derived from C and C++. For the realization of nEMoS, an Arduino UNO shield has been used. Low cost sensors have been installed on the microcontroller in order to detect the environmental variables. The configuration of the overall monitoring device is summarized in Table 1.

**Figure 1:**
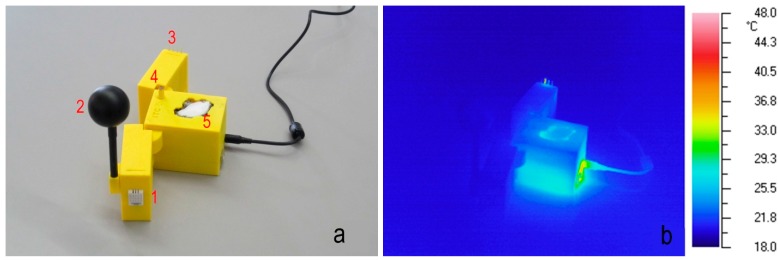
(**a**) Assembled case with electronics; (**b**) Thermographic analysis.

The monitoring system is fitted in an appropriately designed case, so that it can be easily assembled and disassembled. The case was printed with yellow polylactic acid (PLA) and a PowerWasp printer. Figure 1a shows the case and the disposition of the sensors: (1) the air temperature and relative humidity sensor; (2) the globe thermometer; (3) the anemometer; (4) the LDR; (5) the CO_2_ concentration sensor. Some sensors could be affected by thermal effect (for example temperature and/or relative humidity). To rule out this possibility, a thermographic analysis was made. Figure 1b shows the thermographic results: the heat sources are sufficiently away from the sensors.

**Table 1:** Configuration of the monitoring tool.

Purpose	Sensor
Printed circuit board	Arduino UNO with atmel atmega328
Web connection	Arduino Wi-Fi shield
Bluetooth connection	BlueSMiRF Gold
Air temperature & Relative humidity	DHT22
Radiant temperature	10 k thermistor
Air velocity	Wind sensor (Modern Device)
Lighting	LDR sensor
CO_2_ concentration	k-30 sensor (CO_2_meter)

The collected data are sent to a cloud server for the storage through the Wi-Fi shield mounted on the device. Among the numerous available solutions, the free service Xively [19] has been chosen. Xively offers a helpful tool for monitoring and control purposes: it allows real-time graphs and widgets in websites to be embedded, historical data from any public feed to be analysed and processed, real-time alerts to be sent and control devices and various environments to be scripted. The Wi-Fi shield has also a microSD slot: if there is no internet connection it could be used to save data locally simply by uploading a different program. Due to the characteristics of the ATmega328 microcontroller mounted on the Arduino UNO it is not possible to upload a more complex program that can both upload data in the cloud server and save locally into microSD.

**Figure 2:**
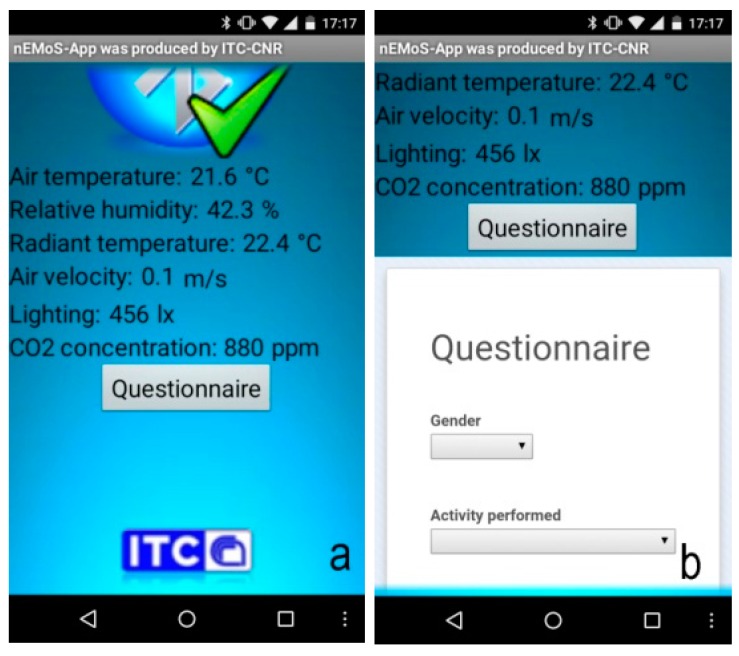
(**a**) nEMoS-App: variables as shown; (**b**) nEMoS-App: first part of the questionnaire.

The hardware architecture includes a Bluetooth module for sending data to smartphones: an app for Android devices was made with the aid of MIT App Inventor, a visual programming blocks language for Android OS [20]. In this way the user has the opportunity to know the thermal comfort conditions (Figure 2a) but also record, through a questionnaire, the personal thermo-hygrometric perception (Figure 2b). By clicking on the send button the data related to the user’s thermo-hygrometric perceptions are stored in a Google spreadsheet. It can be downloaded and used for analysis and comparison with the value of thermal comfort recorded by nEMoS.

## 3. Results and Discussion

A calibration activity was conducted to assess the accuracy of the different sensors, hereinafter referred to a Low Cost sensors (LCs) mounted in the case compared to Professional sensors (Ps). The main difference between the LCs and Ps is related to the calibration certificate which is provided only for the second type. Moreover, the Ps have more exhaustive and complete technical specifications than those of LCs.

### 3.1. Air Temperature and Relative Humidity

Table 2 shows the main characteristics of the sensors used to measure the air temperature and relative humidity. The LCs_DHT22 sensor is connected to Arduino board, while the Ps_UTA sensor is connected to a datalogger of the same manufacturer. In both cases, a single device provides the measurement of the two variables: the Ps_UTA consisting of a 1/3DIN Pt100 sensing element connected with four-wire for air temperature sensing and a thin film that changes the capacity in linear mode with the air humidity.

**Table 2:** Technical specifications sensors used to measure temperature and humidity.

Technical Data	LCs_DHT22	Ps_UTA
Power supply	3.3 ÷ 6 V	10 ÷ 28 V
Typical range	Humidity 0 ÷ 100%RH;	Humidity 0 ÷ 100%RH;
	Temperature −40 ÷ +80 Celsius	Temperature −40 ÷ +85 Celsius
Accuracy	Humidity ±2%RH;	Humidity ±2%RH;
	Temperature < ±0.5 Celsius	Temperature < ±0.1 Celsius
Resolution	Humidity 0.1%RH;	Humidity 0.1%RH;
	Temperature 0.1 Celsius	Temperature 0.015 Celsius
Long-term stability	±0.05%RH/year	-
Response time	Average: 2 s	Average: 8 s
Dimensions	14 × 18 × 4 mm (module)	26 mm (ø) × 220 mm

The LCs_DHT22 sensor has been compared with three Ps_UTA sensors with the same technical characteristics. For the purpose a climate box was used, a calibration device able to recreate a controlled temperature and humidity environment, within a range of established values. The box can operate in a temperature range from −40 °C to +180 °C and it can ensure relative humidity values between 10% and 98%.

The analysis was conducted by setting, on the one hand, a variable temperature profile (Table 3), and on the other hand, a variable relative humidity profile on a fixed number of hours (Table 4), in order to evaluate the sensors in steady-state and transient conditions. Temperature and relative humidity ranges have been chosen in compliance with those possibly detected in the building environment. The two mentioned tables also show the standard deviation of the detected values by the LCs and the Ps for the single ranges.

**Table 3:** Residuals analysis of the temperatures: standard deviation (σ) and average (avg) of the values detected by LCs and Ps related to climatic box

T [°C]	LCs_DHT22		Ps_UTA1		Ps_UTA2		Ps_UTA3	
	σ	avg	σ	avg	σ	avg	σ	avg
5	0.14	0.32	0.15	0.18	0.18	0.13	0.18	0.1
15	0.23	0.32	0.44	0.57	0.45	0.61	0.46	0.53
25	0.07	0.32	0.23	0.78	0.28	0.86	0.27	0.76
35	0.03	0.33	0.19	0.97	0.24	1.10	0.23	0.97

**Table 4:** Residuals analysis of the relative humidity: standard deviation (σ) and average (avg) of the values detected by LCs and Ps related to climatic box

RH [%]	LCs_DHT22		Ps_UTA 1		Ps_UTA 2		Ps_UTA 3	
	σ	avg	σ	avg	σ	avg	σ	avg
30	3.28	3.69	3.40	2.24	3.13	1.01	3.55	−0.08
50	1.29	1.77	2.20	0.17	2.64	−2.17	2.37	−2.75
60	0.68	2.83	0.58	1.15	0.72	−0.45	0.63	−1.70

**Figure 3:**
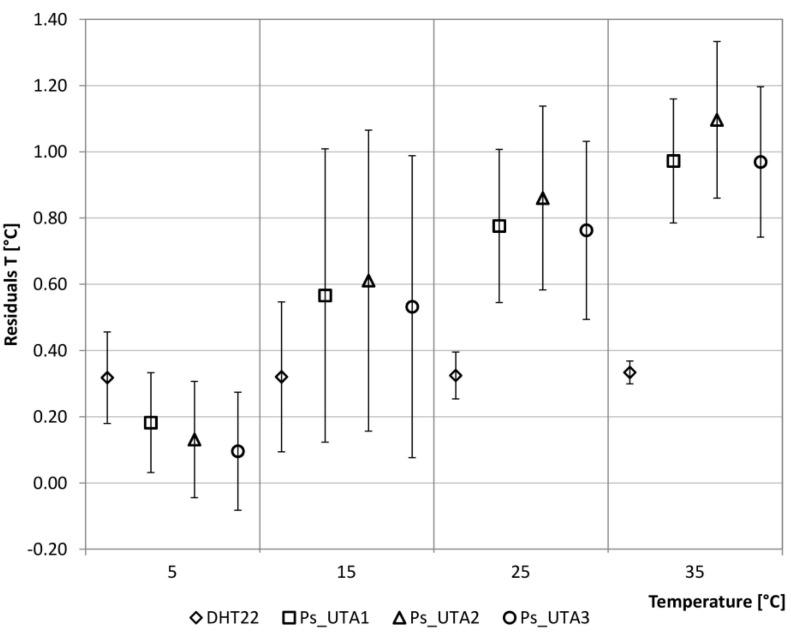
Residuals analysis of the temperatures.

Calibration residuals both for temperature (°C_T_climatic_box_ − °C_T_sensor,i_) and relative humidity (%_RH _climatic_box_ − %_RH_sensor,i_) are considered. The residual analysis of the temperature shows, in general, lower values detected by the considered sensors than the climatic box settings. In more detail, the analysis of the LCs_DHT22 shows how the temperature detected by the sensor tends to be constant and lower than the climate box over the considered range with a mean of 0.32 °C and a maximum standard deviation of 0.23 °C. The other Ps have a more variable trend over the range, especially when the temperature increases: with a set point equal to 5 °C, the detected temperature is very close to the climate box (mean and standard deviation lower than 0.2 °C) and diverges at higher temperatures (Figure 3).

**Figure 4:**
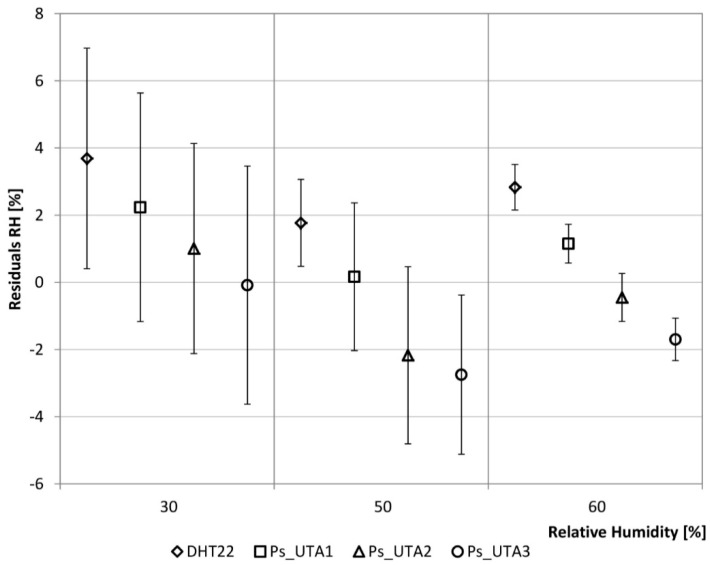
Residuals analysis of the relative humidity.

The relative humidity detected by the LCs_DHT22 is lower than the climate box setting with a maximum difference of about 4% and a the maximum variation of 3.28%, both corresponding to the first range (30% RH). When the set point RH increases the detected values fit the reference data better. In terms of accuracy the Ps have alternately the best behavior, so in terms of precision the LCs_DHT22 is comparable with the Ps (Figure 4). The analysis shows a good behavior of the LCs in detection of temperature and relative humidity and this result makes them suitable for the purpose of the device.

**Figure 5:**
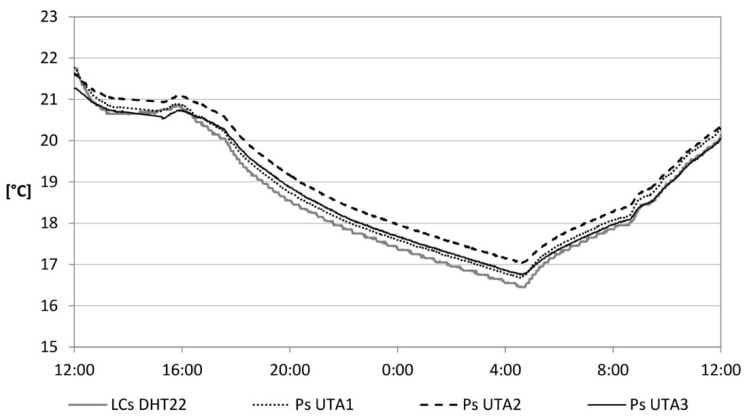
Air temperature.

**Figure 6:**
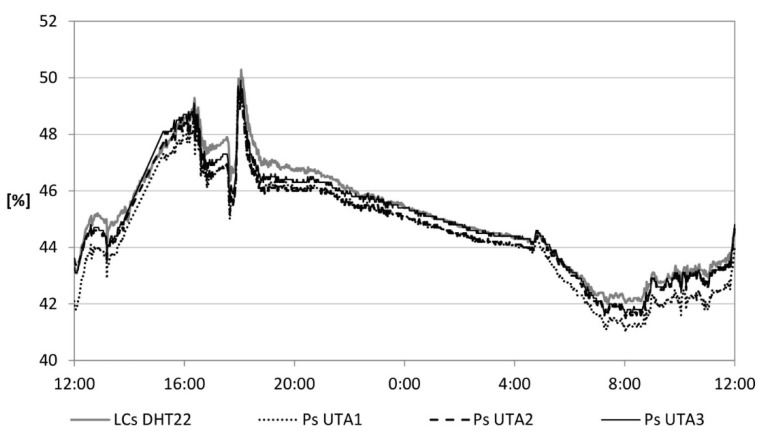
Relative humidity of the air.

Another calibration of the LCs was conducted in a real environment consisting of an office. Ps were also placed within the environment. Data were recorded and sent to the cloud server for a period of 3 days. The trend of the temperature and relative humidity related to the air in a day is shown in Figure 5 and Figure 6, respectively. A percentage difference above 10% is recorded in 3.5% and 5% of cases, respectively. Most data fall within a percentage difference of 5%, in 83% and 72% of cases, respectively.

### 3.2. Radiant Temperature

The measurement of radiant temperature is carried out through a globe thermometer. This device is available in different types, but a spherical object is generally used because it approximates well the volume/surface ratio of the human body. The Vernon globe thermometer consists of a temperature probe inserted in a hollow sphere with a 15 cm diameter. The ball is made of very thin metal and its surface is coated with black diffusing paint. The globe thermometer by Humphreys consists of a mercury thermometer placed inside a sphere with a 40 mm diameter, painted in matt black on the outside [21]. This last solution has two clear advantages: it improves the response times and the portability of the instrument [22].

In replacement of the temperature probe of the Vernon globe thermometer and the Humphreys mercury thermometer, a 10 k thermistor has been used. The characteristics of thermistor are 5 V power, measuring range between −55 and + 60 °C, an accuracy of ±0.2 °C, a response time of less than 10 s and a long-term stability of 0.02 °C/year. The thermistor is connected to the microcontroller through an unshielded rigid cable and inserted inside the 40 mm diameter hollow sphere, painted in matt black, to a depth equal to the radius of the sphere itself.

Like the procedure described for the temperature and relative humidity sensor, a comparison has been conducted between the low cost device (LCs_G) and a professional sensor (Ps_Gx) consisting of a 1/3DIN Pt100 sensing element connected with four-wire, powered by 12 V whose measuring range is between −40 and +60 °C, with an accuracy of ±0.1 °C, a response time of less than 10 s and a resolution of 0.01 °C. In particular, the LCs_G was compared with two professional sensors, having the same technical characteristics (Table 5).

**Table 5:** Technical specifications sensors used to measure radiant temperature.

Technical Data	LCs_G	Ps_G1	Ps_G2
Power supply	3.3 ÷ 5 V	10 ÷ 30 V	10 ÷ 30 V
Typical range	−55 ÷ +60 Celsius	−40 ÷ +60 Celsius	−40 ÷ +60 Celsius
Accuracy	±0.2 Celsius	±0.2 Celsius	±0.2 Celsius
Resolution	-	0.01 Celsius	0.01 Celsius
Long-term stability	±0.02 Celsius/year	-	-
Response time	<10 s	<10 s	<10 s
Dimensions	40 mm (ø)	150 mm (ø)	150 mm (ø)

**Figure 7:**
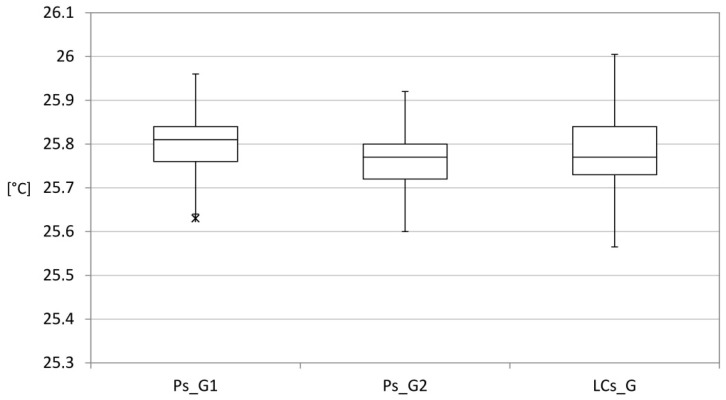
Radiant temperature: indexes of position and variability of the data—Complete Series.

Figure 7 shows the position indices of the data. The values do not show significant differences. All LCs_G readings show a difference less than 2% in relation to both Ps_G1 and Ps_G2.

**Figure 8:**
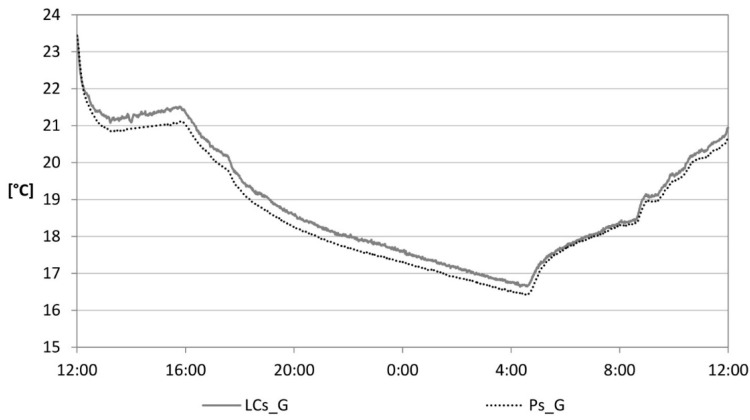
Radiant temperature.

The low cost sensor for the measurement of radiant temperature was also mounted on the device located within the environment under consideration. Data were recorded and sent to the cloud server for a considerable period. Figure 8 shows the comparison with the data recorded by the professional globe thermometer. The values do not show significant differences. LCs_G show a difference of less than 2%, in relation to the Ps_G units.

### 3.3. Air Speed

The air speed in closed environments is normally medium-low and, consequently, the anemometer that better fits this type of measures is the hot-wire anemometer. This technique is based on the heating of an element at a constant temperature and the consequent measurement of the electrical power required to maintain the temperature of the heated element as a function of the air speed change. In this specific case, the Modern Device Wind Sensor (LCs_AS) is considered. This sensor differs from the traditional hot-wire anemometers because it uses a thermistor maintained at a temperature slightly higher than 50 °C.

The reliability of this sensor has been proven by direct comparison with a professional sensor with the aid of a wind tunnel [23]. The developed wind tunnel consisted of the following three main parts: a convergent diffuser, a test chamber and a divergent diffuser.

Both the low cost sensor (LCs_AS) and the professional anemometer (Ps_AS) were inserted in the test chamber. The latter, powered with 9 V, has a measuring range between 0 and 20 m/s, a precision of 0.03 m/s and a resolution of 0.01 m/s (Table 6).

**Table 6:** Technical specifications sensors used to measure air speed.

Technical Data	LCs_AS	Ps_AS
Power supply	4 ÷ 10 V	10 ÷ 30 V
Typical range	-	0 ÷ 20 m/s
Accuracy	-	0.03 m/s
Resolution	-	0.01 m/s
Long-term stability	-	-
Response time	-	-
Dimensions	17.3 × 40.3 × 6.3 mm	182 × 64 × 40 mm

**Figure 9:**
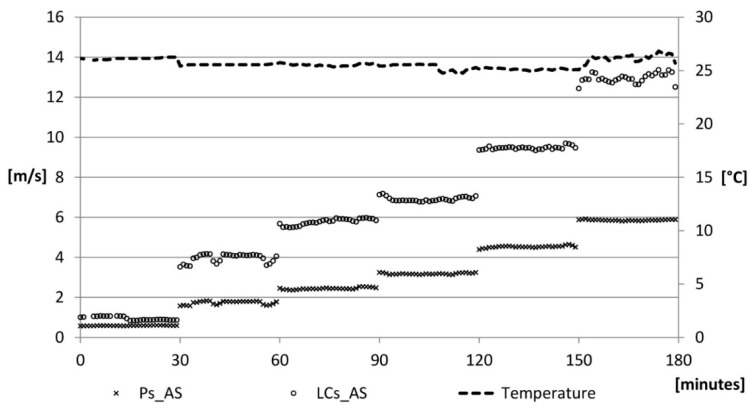
Air speed velocity: verification phase.

As the air velocity increases, the values reported by the LCs_AS diverge, compared to those recorded by Ps_AS (Figure 9).

The algorithm suggested by the manufacturer that determines the air speed is as follows:

(1) $$\text{WindSpeed}\_\text{m}/\text{s}=\frac{\text{WindSpeed}\_\text{MPH}}{2.23694}$$

(2) $$\text{WindSpeed}\_\text{MPH}={\left(\frac{\left(\text{RV}\_\text{WindVolts}-\text{zeroWindVolt}\right)}{0.23}\right)}^{\text{A}}$$

(3) zeroWindVolts=(zeroWindADunits×B)−zeroWindAdjustment

(4) $$\text{zeroWindADunits}=-0.0006\times \left({\text{TMP}}_{\text{ThermADunits}}\times {\text{TMP}}_{\text{ThermADunits}}\right)+\;+\;1.0727\times \text{TMP}\_\text{ThermADunits}+47.172$$

where:
TMP_ThermADunitsis the analogue reading of Pin TMP;RV_WindADunitsis the analogue reading of Pin RV;RV_WindVoltsis given by: RV_WindADunits x B;zeroWindAdjustmentis equal to 0.2;Ais equal to 2.7265;Bis equal to 0.0048828125.

While the coefficient B is the conversion factor given by the ratio between Vin equal to 5 V and 1024 possible values of analogRead, the coefficient A is supplied by the manufacturer. Because of the increasing divergence, for higher and higher air speeds of the LCs_AS values compared to Ps_AS, the A coefficient was modified. In particular, the A^*^ coefficient can be defined as a function of the RV_WindVolts variation with a quadratic function:

(5) $$A^{\text{*}}=-4.089\times \text{RV}\_{\text{WindVolts}}^{2}+22.697\times \text{RV}\_\text{WindVolts}-29.371$$

**Figure 10:**
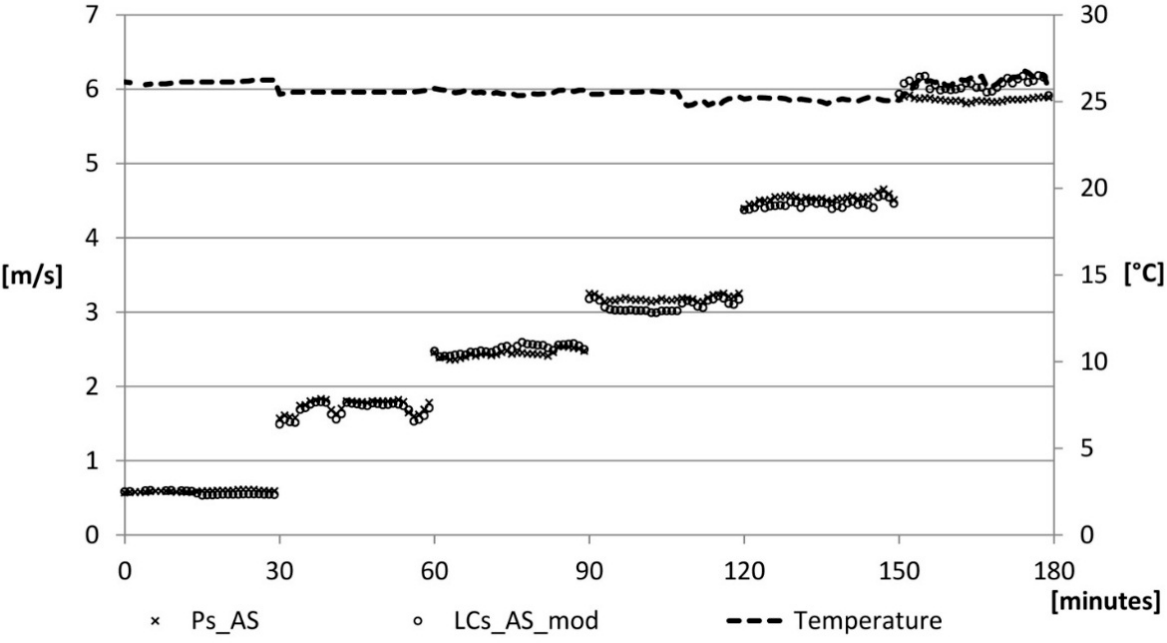
Air speed velocity: correction phase.

The obtained correction factors, A^*^, have been applied to the algorithms provided by the manufacturer which has resulted in comparable values. Low differences between the two sensors have been detected: differences of more than 10% in 3% of the cases (at speeds of the order of 0.5 m/s), and differences of less than 5% in 87% of the cases (Figure 10).

In this case the test was not repeated in the real environment as for the case of sensors for measuring the air temperature, the relative humidity and the radiant temperature because the air speed is susceptible to significant variations depending on the placement of the device in the environment. Table 1 shows the other two sensors: a LDR sensor and a k-30 sensor. The former allows one to evaluate the illuminance over time; the latter is used to determine the level of CO_2_ concentration of the environment. Below are some details.

### 3.4. Lighting

The measurement of the lighting levels of the environment is carried out with the luxmeter. A comparison has been conducted between a low cost luxmeter (LCs_L) consisting of a Light-Dependent Resistor (LDR) powered by 5 V, size 4 × 2 × 5 mm, and a professional luxmeter (Ps_L) with calibration certificate based on a silicon photocell, powered by 3 V, whose measuring range is from 0.01 to 299,900 lx, with an accuracy of ±2% of reading value and dimensions of 69 × 174 × 35 mm (Table 7).

**Table 7:** Technical specifications sensors used to measure lighting.

Technical Data	LCs_L	Ps_L
Power supply	5 V	3 V
Typical range	-	0.01÷299,900 lx
Accuracy	-	±2% of measured value
Resolution	-	0.01 m/s
Long-term stability	-	-
Response time	-	-
Dimensions	2 × 4 × 5 mm	69 ×174 × 35 mm

The sensors have been placed in a dark room, under a lamp with a maximum power light output of 1800 lumens. The illuminance was measured at a distance of 75 cm from the light source so as to guarantee a light cone with a sufficiently large diameter to ensure uniform conditions for the different sensors on the measuring plane. The light source is connected to a potentiometer so as to vary the intensity and to a current stabilizer so as to avoid abrupt voltage changes.

**Figure 11:**
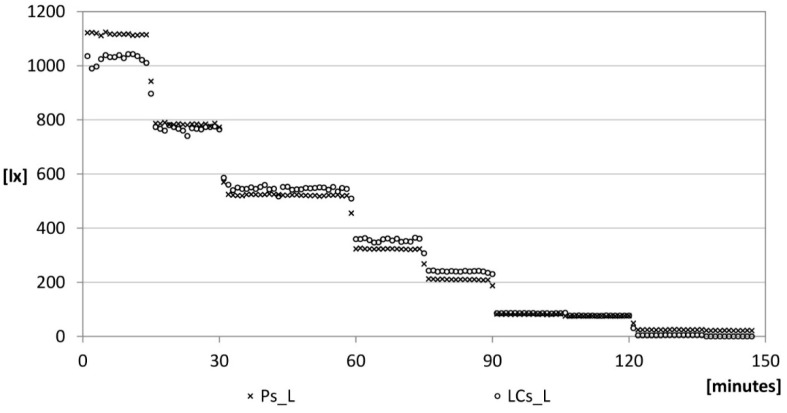
Lighting: verification phase.

After the verification phase, the sensor is mounted on the device and placed within the same experimental set-up to ensure that the returned values were comparable to those of the professional sensor (Figure 11). The LCs_L curves is defined taking into account the Equations (6)–(8):

(6) $$\text{LCs}\_\text{L}={\left(\frac{R}{R_{1}}\right)}^{\left(1/-a\right)}$$

(7) $$\text{R}=\frac{5}{\left(V_{out}\times R_{10k}-R_{10k}\right)}$$

(8) $$\text{Vout}=(\;V_{indv}\times analogread\left(\text{LCs}\_\text{L}\right))$$

where:
Ris the value read by the microcontroller transformed into ΩR_1_is the electrical resistance of the LCs_L sensor in unitary light conditions, initially equal to 94,000 ΩR_10k_is the measure value of the 10k resistor connected to LCs_LAis a value provided by the manufacturer, known as Gamma valueV_indv_is the conversion factor equal to 0.004882812, given by the ratio between V_in_ = 5 V and 1024, a possible values of analogRead (LCs_L)

It was decided to modify the coefficient R_1_: for each single step of luminous intensity the optimal value to reduce the differences for lighting intensity in the range 0–700 lx was identified. Afterwards the performance approximated equation as a function of the variable analogRead (LCs_L) was defined:

(9) $${R_{1}}^{\text{*}}=0.7464\times \text{analogRead}{\left(\text{LCs}\_\text{L}\right)}^{2}-1220\times \text{analogRead}\left(\text{LCs}\_\text{L}\right)+582596$$

**Figure 12:**
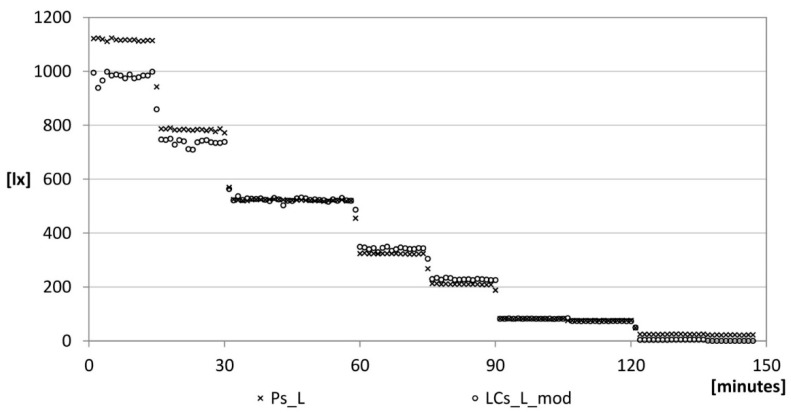
Lighting: correction phase.

By replacing *R*_1_^*^ in Equation (6), the trend shown is Figure 12 is obtained.

It can be noted that the LDR mounted on the device gives acceptable values for illuminance values lying between 40 and 800 lx. The detected differences between the two sensors are more than 10% in 5% of the cases.

### 3.5. CO_2_ Concentration

Carbon dioxide is an odorless and tasteless gas, perceivable only due to its negative effects: illness, concentration difficulties and poor performance. For this measurement, the K-30 sensor of the CO_2_meter is used, whose measurement range is 0 to 10,000 ppm with an accuracy of 3% and a repeatability of 1% compared to the measured value. The behavior of this sensor has not been compared with a commercial tool.

## 4. Conclusions and Future Work

The article summarizes the implementation phases of a device aimed at monitoring the environmental variables of a confined space, through the use of low cost sensors and open source hardware/software. The choice of optimal LC devices was performed by evaluating the economic and consistency conditions of the collected data. The performed analyses have shown that the values measured by the chosen sensors have only minor percentage differences compared to those of commercial tools, in most cases, less than 5%. It should be noted that the comparison has been carried out under controlled conditions. The nEMoS’ features make it useful in confined spaces where little invasiveness and reduced installation time are needed. It has already been successfully used to assess the thermal comfort in a nursing home and offices.

The future development of the nEMoS consists of different steps: first of all it’s necessary to complete the comparison of CO_2_ concentration values acquired by the K-30 sensor with those obtained from other professional sensor; another update is the substitution of the LDR for measurement of the lighting levels of the environment with some other cheap sensor: the TSL2561 could be a good upgrade because it is able to detect a wider light range from 0.1 lx to 40 klx.
